# Adhesive Films Based on Benzoxazine Resins and the Photoreactive Epoxyacrylate Copolymer

**DOI:** 10.3390/ma15051839

**Published:** 2022-03-01

**Authors:** Agnieszka Kowalczyk, Marta Tokarczyk, Mateusz Weisbrodt, Konrad Gziut

**Affiliations:** Department of Chemical Organic Technology and Polymeric Materials, Faculty of Chemical Technology and Engineering, West Pomeranian University of Technology in Szczecin, 70-322 Szczecin, Poland; tm43148@zut.edu.pl (M.T.); mateusz.weisbrodt@zut.edu.pl (M.W.); konrad.gziut@zut.edu.pl (K.G.)

**Keywords:** benzoxazine resins, structural adhesives, epoxyacrylate copolymers, polymer blends, UV-cross-linking, adhesion

## Abstract

UV-cross-linkable and thermally curable self-adhesive structural tapes (SATs) were compounded using solid commercial benzoxazine resins (Araldite MT 35700 and Araldite MT 35910) and a photoreactive epoxyacrylate copolymer (EAC). As initiators of benzoxazine resin polymerization and epoxy component cationic polymerization, two kinds of latent curing agents (LCAs) were tested, i.e., amine type and ionic liquid type. The influence of the benzoxazine resin and the LCA type on the UV-cross-linking process, the self-adhesive features and thermal curing behavior of UV-cross-linked tapes, as well as the shear strength of cured aluminum/SAT/aluminum joints and thermal stability of adhesives were investigated. It was found that the amine additive and the benzoxazine resin take part in the UV-cross-linking process of the EAC as hydrogen donors, which is confirmed by an increase in cohesion (+86%) and a decrease in adhesion (−25%) of SATs. The highest results of adhesion to steel (47 N/25 mm) and overlap shear strength (11.1 MPa) values were registered for SATs based on Araldite MT 35910 and contained 7.5 wt. parts of the amine-type hardener. The formation of a polyacrylate-benzoxazine network has a significant impact on the course of the thermal curing process and the thermomechanical properties of adhesive joints, which was also confirmed by the Cure Index calculation.

## 1. Introduction

Benzoxazine resins (BRs) are a novel class of thermosetting phenolic resins that have a characteristic oxazine ring (a heterocyclic six-membered ring with oxygen and nitrogen atoms) [1]. They can by synthesized via the Mannich condensation reaction of a phenolic derivative, formaldehyde, and a primary amine [2]. In 1988, Turpin and Therane described the preparation of small-molecular-weight BRs from multifunctional phenols and multifunctional amines [3]. Due to the great interest in the use of BRs, new “green” methods of obtaining them have been proposed as well [4,5]. The polymerization of BRs typically takes place through thermally accelerated ring-opening polymerization (ROP), which occurs with or without initiators, and no by-products are produced. The polymerization of BRs takes place by heating (between 160 and 220 °C) because BRs typically contain a small amount of phenolic derivatives (raw material) as impurity [6]. It has been reported that some acids (e.g., adipic acid), bases, and Lewis acid can effectively decrease the induction for benzoxazine polymerization [7,8,9]. The reaction with a labile proton initiator (such as a phenol) leads to the polymer with a phenolic structure. If a nonlabile proton initiator (such as the Lewis acid) acts, the arylether structure is formed. Moreover, the characteristic oxazine rings can be used to form a cross-linking network. Polybenzoxazines have outstanding properties, such as high glass transition temperature, high thermal stability, low water absorption, and excellent mechanical performance [10,11,12]. Therefore, they have a huge research and industrial application potential for various purposes, e.g., as components of self-healing systems (smart materials) [13]; as materials for space radiation shields [4]; as materials for aerospace application [14]; and as coatings, sealants and adhesives and others [15,16,17,18,19,20,21,22,23,24,25,26,27,28]. 

The ability of BRs to react with other resins, e.g., epoxy resins (ERs), to produce hybrid formulations with unique properties is their advantage as well. Schreiber et al. first reported BR-ER systems in the early 1970s [29]. It was revealed that the polymerization reaction of BR-ER mixtures shows two exothermic peaks; the first peak, at a lower temperature, was assigned to the exothermic polymerization of the BR neat, and the second peak was assigned to the reaction of the ER using the BR as a hardener [30,31]. However, BRs also have their disadvantages, i.e., a high curing temperature, limited processability (they are mostly powder), and difficulties in film preparation. Nowadays, blending is considered as one of the methods to improve the thermal and mechanical properties as well as the processability of benzoxazine resins [32]. For this reason, it seemed interesting to prepare adhesive films (i.e., self-adhesive structural tapes (SATs)) based on selected benzoxazine resins. 

SATs are a special kind of adhesives, i.e., they are tacky at room temperature during application to the surface (metals, ceramics) and they have a relatively long pot-life (which determines their suitability for use). In this respect, they behave like typical pressure-sensitive adhesives. However, after joining elements (thermally curing process), they form adhesive joints with high shear strength (according to ASTM > 7 MPa) [33,34]. As a result, SATs can be classified as structural adhesives. A new type of SATs, containing benzoxazine resins (in place of the commonly used epoxy resins) and a photoreactive epoxycopolymer (EAC) has been presented. Generally, the influence of BR types and the latent curing agent kind (amine type or ionic liquid type) on the course of the UV-cross-linking process of SATs and the (thermo)mechanical features of adhesive films were tested and are discussed in detail. Additionally, a comparative evaluation of the effectiveness of the thermal curing process of the epoxyacrylate copolymer (EAC) against benzoxazine resins or classic epoxy resin (i.e., Cure Index) was performed. Finally, the adhesive, mechanical, and thermal properties of SATs based on benzoxazine resins were compared to those based on epoxy resin.

## 2. Materials and Methods

### 2.1. Materials

**Figure 1:**
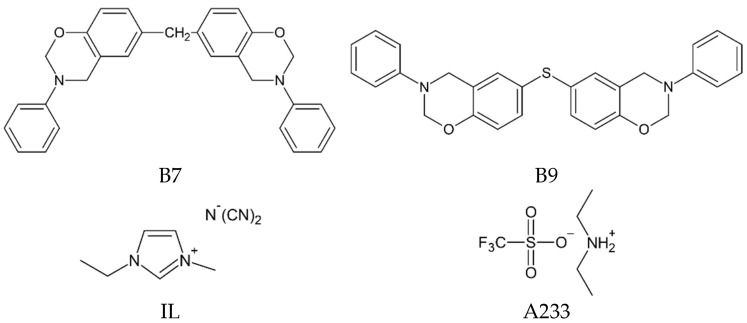
Chemical structures of tested benzoxazine resins and latent curing agents: B7—benzoxazine resin Araldite MT 35700; B9—benzoxazine resin Araldite MT 35910; IL—1-ethyl-3-methylimidazolium dicyanamide; A233—diethylamine salt of trifluoromethanesulfonic acid.

The following components were used for the preparation of epoxyacrylate copolymers (EAC): n-butyl acrylate (BA); glycidyl methacrylate (GMA); 4-hydroxybutyl acrylate (HBA) (BASF, Ludwigshafen, Germany); 4-acryloyloxy benzophenone (ABP, Chemitec, Scandiccy, Italy); 2,2-azobis(isobutyronitryle) (AIBN, Merc, Darmstadt, Germany); and ethyl acetate (POCh, Gliwice, Poland) as a solvent. The commercial benzoxazine resins Araldite MT 35910 and Araldite MT 35700 (Huntsman, Everberg, Belgium), latent curing agents (IL-0003-HP, Ionic Liquids Technologies GmbH, Heilibronn, Germany; and Nacure Super A233, Worleé Chemie, Hamburg, Germany), a multifunctional monomer (Laromer 9023, BASF, Germany), a radical photoinitiator (Omirad 127, IGM Resins, Waalwijk, The Netherlands), and a surface-tension-reducing compound to improve wettability of the substrates (Byk 325, Byk-Chemie, Wesel, Germany) were used as components of the self-adhesive structural tapes (SATs). The chemical structures and characteristics of benzoxazine resins (BRs) and latent curing agents (LCAs) are showed in Table 1 and Figure 1, respectively.

### 2.2. Preparation and Characterization of Epoxyacrylate Copolymer (EAC)

The EAC was synthesized via free radical bath copolymerization of BA (7.99 mol), GMA (1 mol), HBA (1 mol), and ABP (0.01 mol) in ethyl acetate using AIBN as an initiator (9.1 wt. part/100 wt. parts of the monomers). The copolymerization process was realized at 78 °C for 5 h in a glass reactor equipped with a mechanical stirrer. The prepared product (i.e., copolymer solutions) contained 50 wt.% of solids. 

Dynamic viscosity of the EAC solution and gel permeation chromatography measurements were performed as previously reported [35]. The characteristic of the EAC are shown in Table 2.

### 2.3. Preparation and Characterization of Adhesive Compositions and Structural Self-Adhesive Tapes (SATs) Based on Benzoxazine Resins 

Adhesive compositions compounded by using BRs (50 wt. parts), the EAC solution (100 wt. parts), and the latent curing agent (2.5 wt. part of A233 or IL) were tested in respect of their pot-life and thermal properties (thermal curing process). The viscosity change with time was a measure of the pot-life. The increase in the viscosity of samples was assessed by a visual inspection of the sample (10 mL) flow in the glass vial (20 mL). A thermal analysis (i.e., a thermal curing process) using the differential scanning calorimetry method (DSC Q100, TA Instr., New Castle, DE, USA) was performed. The glass transition temperature (T_g_), the enthalpy of curing processes (ΔH), the onset temperature of the curing reactions (Ti), and the maximum temperature of the curing reaction (T_p_) were examined. Samples (ca. 10 mg) were analyzed using hermetic aluminum pans in the temperature range of −80–350 °C (heating rate of 10 °C/min).

Structural self-adhesive tapes (SATs) were compounded using BRs (50 wt. parts), the EAC solution (100 wt. parts), the latent curing agent (2.5, 5, or 7.5 wt. parts), and BYK 325 (0.75 wt. part). Moreover, Laromer 9023 (2 wt. parts) and Omnirad 127 (1 wt. part) were added in order to increase the cohesion of SATs after UV cross-linking. A description of the prepared SATs is provided in Table 3. The components were homogenized at 23 °C for 15 min (500 rpm) by the RE16 mechanical mixer (IKA, Staufen, Germany). Then, the compositions were applied onto polyester foil (samples for self-adhesive tests) or siliconized paper (other tests), dried at 105 °C for 10 min, and UV irradiated using the medium-pressure mercury lamp (UV-ABC, Hönle UV-Technology, Gräfelting, Germany). The UV exposition was controlled with a radiometer (Dynachem 500, Dynachem Corp., Westville, IL, USA). The base thickness of the UV-cross-linked SATs was 100 µm. After the UV-cross-linking process, the fundamental properties of SATs (i.e., self-adhesive properties) were tested according to Association des Fabricants Europe’ens de Rubans Auto-Adhe’ sifs (AFERA) standards, i.e., AFERA 5001 (adhesion to a steel substrate), AFERA 5015 (tack), and AFERA 5012 (cohesion). Adhesion is defined as the force value required to remove a pressure-sensitive material from a stainless steel plate; the removal proceeds at an angle of 180° at a speed of 300 mm/min. Tack is characterized by a force value required to separate a stainless steel plate and an adhesive tape applied under low pressure for 0.5 s. Cohesion (i.e., static shear adhesion) describes the time needed to shear off the adhesive tape sample (under a load of 1 kg) from a defined steel surface. These parameters were evaluated using five samples of each adhesive tape. 

### 2.4. Preparation and Characterization of Al/SAT/Al Joints and Thermally Cured SATs

Aluminum/SAT/aluminum overlap joints (Al/SAT/Al) were prepared using the UV-cross-linked SATs and degreased 2024 aluminum panels (100 mm × 25 mm × 1.6 mm); the joints were thermally cured at 180 °C for 60 min, 195 °C for 60 min, or 195 °C for 90 min. The shear strength of the Al/SAT/Al systems was measured at room temperature according to the ASTM D1002-10 standard (10 samples of each system) using the Z010 machine (Zwick/Roell, Ulm, Germany). Additionally, the cross-linking degree (*α*) of the thermally cured SATs was calculated using DSC data according to Equation (1) [36].

(1) $$\alpha =\left(\frac{\Delta H_{T\;}-\Delta H_{res}}{\Delta H_{T}}\right)\;(a.u.)$$

where Δ*H_T_* is the total enthalpy of the SAT curing process (J/g) and Δ*H_res_* is the enthalpy of the post-curing process of the thermally cured SAT (in an Al/SAT/Al joint). 

The thermal stability of the thermally cured SATs (in an N_2_ atmosphere) was measured by using the TG Libra Analyzer (Netzsch, Selb, Germany). Samples (ca. 10 mg) were heated in aluminum pans at the temperature range of 25–1000 °C (10 °C/min). The initial decomposition temperature, at which 5% of the material has evaporated (T_d5_), and the temperature of the loss of 50% of the sample mass (T_d50_) were determined. 

The Cure Index (CI) for evaluating the status of the curing reaction in thermoset composites, i.e., EAC/BRs or with bisphenol-A-based liquid epoxy resin (EAC/ER), based on experimental data on nonisothermal DSC, according to Equations (2)–(4), was determined [37].

(2) $$CI=\Delta H^{*\;}\cdot \Delta T^{*}$$

(3) $$\Delta H^{*}=\frac{\Delta H_{C}}{\Delta H_{ref}}$$

(4) $$\Delta T^{*\;}=\frac{\Delta T_{C}}{\Delta T_{ref}}$$

where Δ*H_C_* and Δ*H_ref_* are the total heat values released during the cure reaction of thermoset systems (SAT-B7 or SAT-B9) and the reference system (SAT-0 based on ER, according to [35]), respectively, and Δ*Tc* and Δ*T_ref_* are the cure temperature intervals for thermoset systems (SAT-B7 or SAT-B9) and the reference sample (SAT-0), respectively. 

## 3. Results

**Figure 2:**
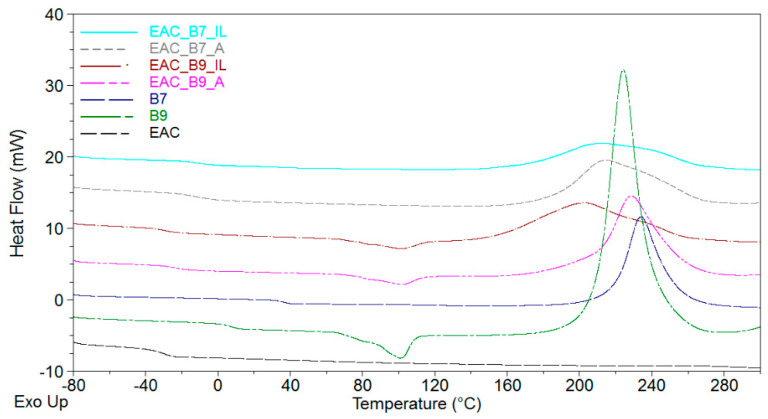
DSC thermographs for an epoxyacrylate copolymer (EAC), benzoxazine resins (B7 and B9), and the composition of the epoxyarylate copolymer with benzoxazine resins and the latent curing agent (2.5 wt. parts).

The thermal analysis and the pot-life of adhesive compositions containing only an epoxyacrylate copolymer, a benzoxazine resin (EAC-BR; 100/50 wt. parts.), and 2.5 wt. parts of the LCA was performed. Results are shown in Table 4 and Figure 2.

The pot-life of adhesive compositions is a simple but important criterion for their suitability for use. The research shows that samples with the B7 resin, generally, have a longer pot-life than those with the B9 resin. The shortest pot-life was observed for the sample with the ionic liquid (EAC-B9-IL; only 17 days). Such results indirectly indicate the higher reactivity of this system. This is confirmed by the DSC results; values of the onset temperature of the curing reactions (Ti) for samples EAC-B7-IL and EAC-B9-IL are the lowest (121 °C and 117 °C, respectively). 

Moreover, values of the maximum temperature of the curing reaction (T_p_) and the exothermic effect (ΔH) of the reaction initiated by the ionic liquid are lower than those initiated by the amine-type hardener. However, it should be noted that the curing processes in EAC-B7 systems were less exothermic (ca. 170 J/g) than those in the EAC-B9 systems (ca. 200 J/g or more). This is related to the polymerization reaction of benzoxazine resins themselves.

Our research indicates that during the polymerization without the initiator, more heat was generated in the B9 than in the B7 resin (391 J/g and 345 J/g, respectively; Table 4). The enthalpy values of the curing processes in EAC-B7 and EAC-B9 systems are lower than the enthalpy values for pure B7 or B9 resins because in adhesive systems, resins are 50% by weight. The remaining part of the adhesive compositions is the epoxyacrylate copolymer (EAC), which also reacts by the cationic polymerization initiated by the same LCA. It is important to note that the polymerization of BRs themselves begins at temperatures above 200 °C (Table 4). However, in EAC-BR systems with LCAs, the polymerization process starts at significantly lower temperatures (117 °C for EAC-B9-IL and 121 °C for EAC-B7-IL) and for systems with the amine-type hardener at ca. 140 °C.

**Figure 3:**
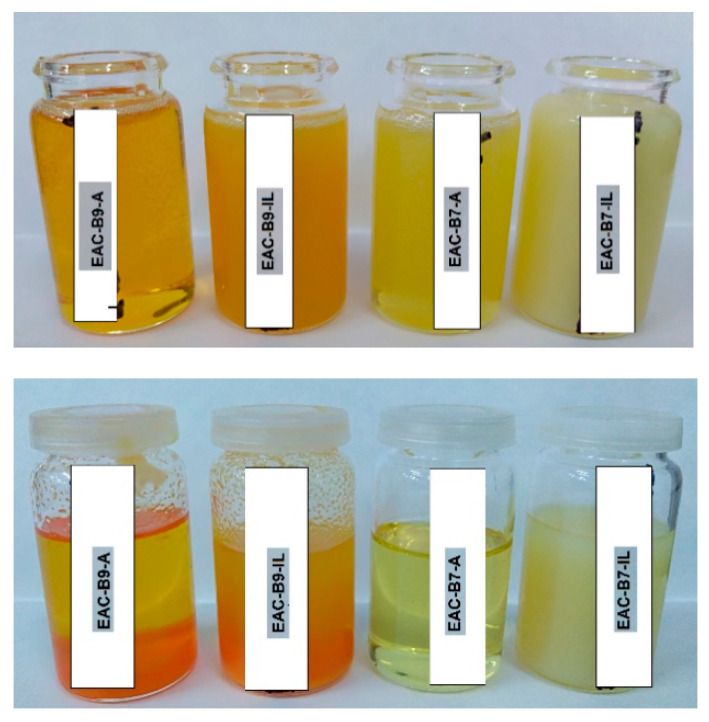
Images of EAC-BR compositions with LCAs right after the homogenization process (panel **above**) and after 3 days (panel **below**).

Exothermic peaks for EAC-BR systems are wide (or overlapping), which might indicate that the polymerization processes (of the epoxy component and the benzoxazine resin) proceed in two stages. DSC studies have also shown that the addition of the BR significantly increases the glass transition temperature (T_g_) of new adhesive systems. It is generally known that adhesive films characterized by a T_g_ higher than −20 °C exhibit unsatisfactory self-adhesive features. Due to the significantly higher T_g_ value of the B7 resin (37 °C) than the B9 resin (8 °C), adhesive systems with the B7 are characterized by higher T_g_ values (−11 °C and −12 °C). Therefore, it is likely that EAC-B9 samples will show better self-adhesive properties. Additionally, DSC thermograms for the sample B9 and its mixtures with the EAC show a characteristic effect around 100 °C, i.e., the melting point of the B9 resin. Both resins are solid at room temperature. However, in the composition with the EAC and after the homogenization step, adhesive systems appear homogeneous. Nevertheless, after several hours, the sedimentation effect can already be observed in EAC-B9-IL and EAC-B9-A systems. This effect is shown in Figure 3.

**Figure 4:**
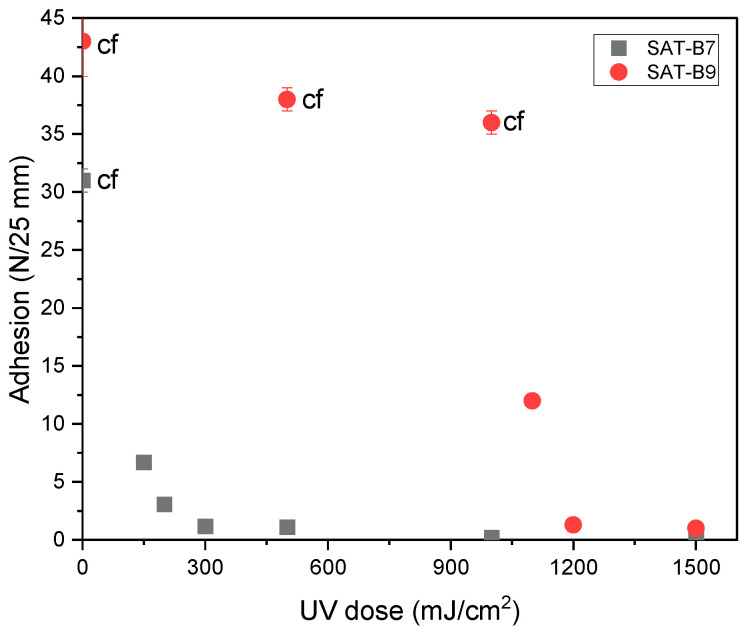
Adhesion to steel of SATs in relation to the UV-cross-linking dose (cf—cohesive failure).

It is interesting that only EAC-B7-A is homogeneous (even after a longer time) and samples with the ionic liquid are cloudy. This may indicate the difficult miscibility of the epoxyacrylate copolymer with benzoxazine components in the presence of the ionic liquid. Although the relatively high T_g_ of systems with the B7 resin could exclude it from further research (expected low adhesion), it is known that BRs may show increased adhesion to the metal substrate due to their structure and the presence of hydrogen bonds [1]. Therefore, further studies were carried out with the use of the mentioned resin as well. Two series of SATs containing different benzoxazine resins, i.e., B7 (SAT-B7) and B9 (SAT-B9) and the latent curing agent (amine type or ionic liquid type) were produced. As a result, 12 SATs were obtained. First, the effect of the UV dose on the adhesion to steel of SAT films based only on the EAC, B7, or B9 resins and Byk 325 (without the cross-linking monomer and the LCA) was determined in order to designate the optimal UV-cross-linking conditions. Results are shown in Figure 4. 

The UV-cross-linking process in SATs is possible because the EAC contains photoreactive benzophenone groups (from type II photoinitiator, i.e., ABP). The mechanism of cross-linking of ABP-containing polyacrylate chains is known from the literature [38]. As can be seen, both SATs before the UV-cross-linking process show high adhesion to steel (32 N/25 mm for SAT-B7 and 43 N/25 mm for SAT-B9) but the cohesive failures effects are visible. In both cases, the adhesion to steel decreases with an increase in the UV dose due to the progressive process of cross-linking of epoxyacrylate chains in the EAC. Relatively high adhesion without the effect of cohesive failure was obtained for SAT-B7 (7 N/25 mm) after irradiation at a low UV dose (150 mJ/cm^2^). A further increase in the UV dose resulted in a significant decrease in adhesion of SAT-B7. However, high adhesion to steel of SAT-B9 (12.5 N/25 mm; without cohesive failure) was obtained after cross-linking at a much higher UV dose (1100 mJ/cm^2^). Arguably, this result is due to the lower dispersion of the B9 resin in the EAC matrix, which makes the UV-cross-linking process of epoxyacrylate chains difficult and requires a higher UV dose (longer irradiation time). It is also noticeable that the adhesion of SAT-B9 films was generally higher than that of SAT-B7 films, as mentioned before. It is caused by the lower T_g_ value of the B9 resin itself (8 °C; Table 4). In summary, all SAT-B7 films were cross-linked at the UV dose of 150 mJ/cm^2^ and SAT-B9 films at 1100 mJ/cm^2^ in the next step.

**Figure 5:**
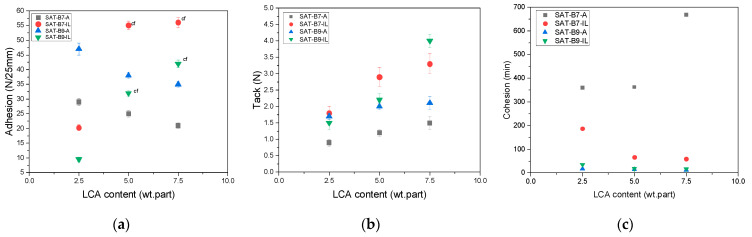
Adhesion to steel (**a**), tack (**b**) and cohesion (**c**) of SATs in relation to the type and content of the latent curing agent (cf-cohesive failure).

Results of the self-adhesive properties (i.e., adhesion to steel, tack, and cohesion at 20 °C) of SATs (with a cross-linking monomer and a type I photoinitiator) in relation to the LCA content (2.5; 5 or 7.5 wt. parts) are presented in Figure 5. At the lowest LCA dose (2.5 wt. part), SAT films show high adhesion (from 9.5 N/25 mm for SAT-B9-IL to 47 N/25 mm for SAT-B9-A) and they are free from failure. It is important to note that adhesion values for SATs vary depending on the type of hardener. As can be seen, for SATs with ionic liquid, adhesion values increase with the increasing amount of this hardening agent. However, the addition of IL strongly hinders the UV-cross-linking process (cohesive failure of films has been reported), regardless of the BR type. As shown above (Figure 3), addition of the IL to the EAC-BR system reduces the miscibility of these components.

**Figure 6:**
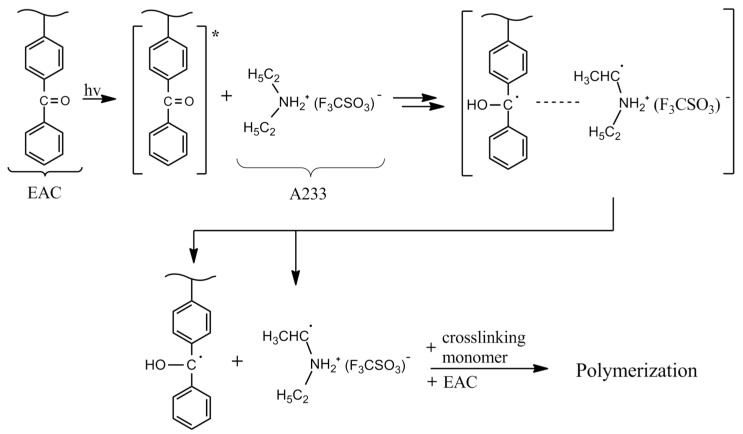
Photoinitiated free radical polymerization of the EAC and the cross-linking monomer (Laromer 9023) using the amine salt as the hydrogen donor.

Additionally, it is known from literature that some of the ionic liquids act as plasticizers [39]. Therefore, their presence in the photoreactive system interferes with the UV-cross-linking process. The addition of an amine-type hardener has the opposite effect: as the content of the A233 increases, the adhesion of SATs decreases, from 29 N/25 mm to 21 N/25 mm for SAT-B7 and from 47 N/25 mm to 35 N/25 mm for SAT-B9. This additive increases the efficiency of the UV cross-linking and the formation of a denser polyacrylate network. Thus, a decrease in the adhesion of SATs is observed. Probably a diethylamine salt of a trifluoromethanesulfonic acid (A233) acts similar to the hydrogen donor of the UV-cross-linking process. Benzophenone-amine photoinitiation systems are known from the literature [40]. The proposed mechanism of the EAC cross-linking with the participation of an amine is shown in Figure 6.

**Figure 7:**
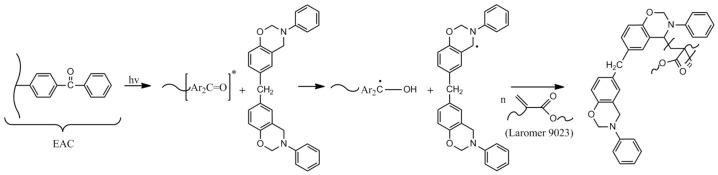
Photoinitiated free radical polymerization using the benzoxazine resin as the hydrogen donor.

Test results showed that the tack of SATs increases with the addition of the LCA for all samples. However, tack values are generally low. For properly cross-linked SATs (without cohesive failure), the range of tack is from 0.75 to ca. 2 N. Higher tack values were recorded only for SATs with a higher ionic liquid content (5 or 7.5 wt. parts). The high adhesion of SATs and low tack result from the presence of benzoxazine resins. Their high T_g_ values affect the high T_g_ of SAT films, causing low tack (lower mobility of epoxyacrylate chains and benzoxazine monomers, which results in poorer wettability of the test plate). Therefore, the high adhesion of SATs seems impressive. First photo-cross-linking tests (Figure 4) concerned only EAC-BR systems, where it is known that the photo-cross-linking could only take place with the participation of benzophenone groups embedded in the EAC chain. Results shown in Figure 5 refer to complex systems (containing the cross-linking monomer and the type I photoinitiator). So far, the possibility of the UV-cross-linking involving the BR has not been considered. It is suspected that in a homogeneous system (SAT-B7-A), the UV-cross-linking process may take place with the participation of the B7 resin. This may be confirmed by the significant increase in the cohesion value of SAT-B7-A (even by 86% for the SAT with 7.5 wt. parts of the A233) due to the cross-linking density in a poly(epoxyacrylate) - benzoxazine network. The proposed reaction scheme is shown in Figure 7. 

It is known that benzoxazine was used as the hydrogen donor in the type II photoinitiating system [1]. In our research, this system consists of poly(epoxyacrylate) chains with a hanging moiety of the aromatic carbonyl group (–Ar_2_C=O) derived from the ABP molecule. The initiating action of the benzoxazine is based on the intermolecular reaction of the excited photosensitizer (–Ar_2_C=O*) with the oxazine ring and the subsequent hydrogen abstraction reaction. Resulting radicals can initiate the polymerization of the cross-linking monomer (acrylic monomer, Laromer 9023). However, during the photopolymerization process (preparation of UV-cross-linked SAT films), the benzoxazine ring structure is conserved and may undergo a subsequent thermal ring-opening reaction (preparation of thermally cured SATs in Al/SAT/Al joints).

**Figure 8:**
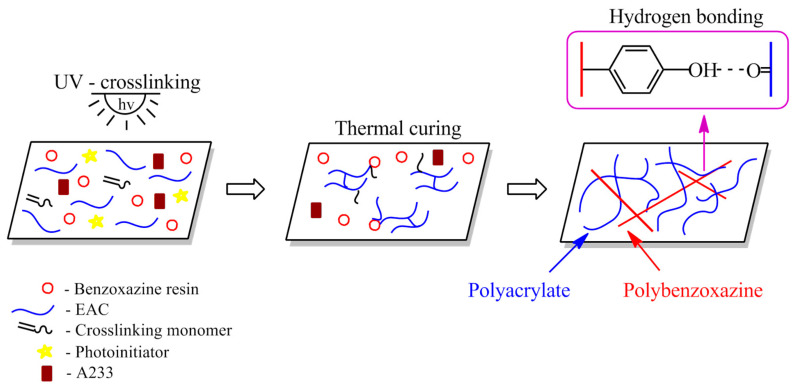
Preparation steps of UV-cross-linked SATs and thermally cured SATs (Al/SAT/Al joints).

The preparation steps of SATs and Al/SAT/Al joints are shown in Figure 8. 

**Figure 9:**
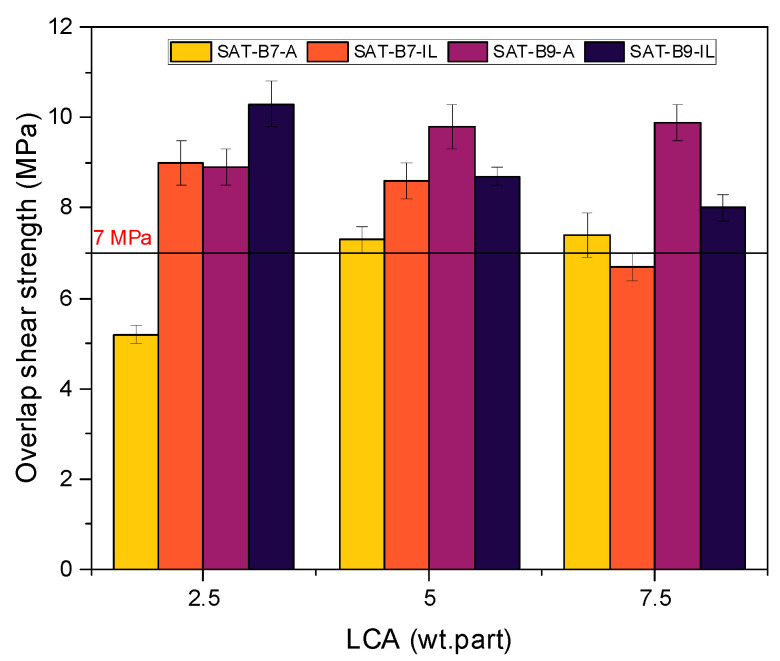
Overlap shear strength of Al/SAT/Al joints (after thermal curing at 180 °C for 60 min) in relation to the latent curing agent type and content in SATs.

UV-cross-linked SATs were applied between aluminum panels and thermally cured at 180 °C for 60 min. Overlap shear strength (τ) values recorded for thermally cured Al/SAT/Al joints are presented in Figure 9. 

**Figure 10:**
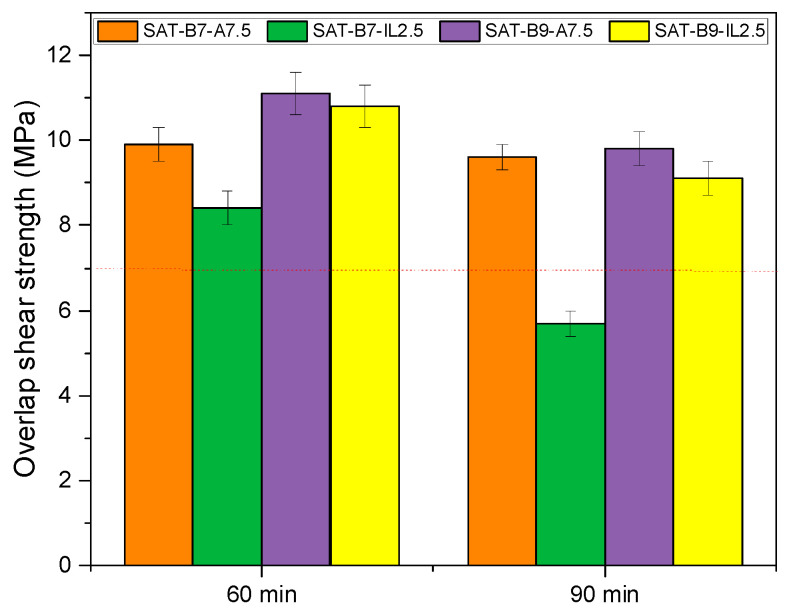
Overlap shear strength of Al/SAT/Al joints after thermal curing at 195 °C for 60 or 90 min.

Most of the τ values were higher than the value required for structural adhesives according to the ASTM standard (7 MPa), except for the cases of SAT-B7-A-2.5 and SAT-B7-IL-7.5 (5.2 MPa and 6.7 MPa, respectively). Overlap shear strength values depend on the type and amount of the LCA as well as the type of the benzoxazine resin. In detail, a higher amine hardener concentration increased τ values: from 5.2 to 7.4 MPa (SAT-B7-A) and from 8.9 to 9.9 MPa (SAT-B9-A). In the case of the use of an ionic liquid hardener, the tendency was opposite; with the increase in the IL, τ results decreased (from 9 to 6.7 MPa for SAT-B7 and from 10.3 to 8 MPa for SAT-B9). Additionally, it can be seen that τ values for the SAT with the B7 resin are in the range from 5.2 to 9 MPa and for samples with the B9, they are higher (8–10.3 MPa). Test results showed that to obtain high τ values, only 2.5 wt. parts of the ionic liquid or 7.5 wt. parts of amine hardener should be used. Based on the above tests, four SATs with the highest τ values were selected, namely SAT-B7-A-7.5, SAT-B7-IL-2.5, SAT-B9-A-7.5, and SAT-B9-IL-2.5. Next, Al/SAT/Al joints were thermally cured at 195 °C for 60 or 90 min in order to check whether thermal curing at a temperature close to the T_p_ (Table 2) will give better τ results. The overlap shear strength effects are shown in Figure 10.

**Figure 11:**
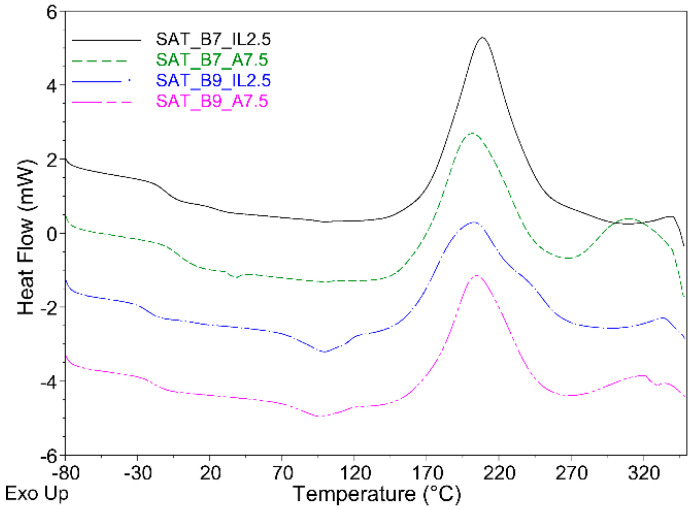
DSC thermograms of UV-cross-linked SATs.

Increase in the curing temperature to 195 °C (while maintaining the same curing time) resulted in an increase in τ values for adhesive joints with the amine hardener (from 5.2 to 9.9 MPa for SAT-B7-A and from 9.9 to 11.1 MPa for SAT-B9-A). In the case of the ionic liquid hardener, only a slight increase (from 10.3 to 10.8 MPa for SAT-B9-IL) or decrease in τ values (from 9.0 to 8.4 MPa for the SAT B7-IL) was recorded. In turn, the extension of the hardening time at 195 °C to 90 min resulted in a decrease in τ values of adhesive joints (in each case). It is known that the shear of structural adhesives depends on their cross-linking degree, and an optimal range of the latter parameter can be often observed. The cross-linking degree (α) and DSC analyses of thermally cured SATs are presented in Figure 11 and Table 5. 

**Figure 12:**
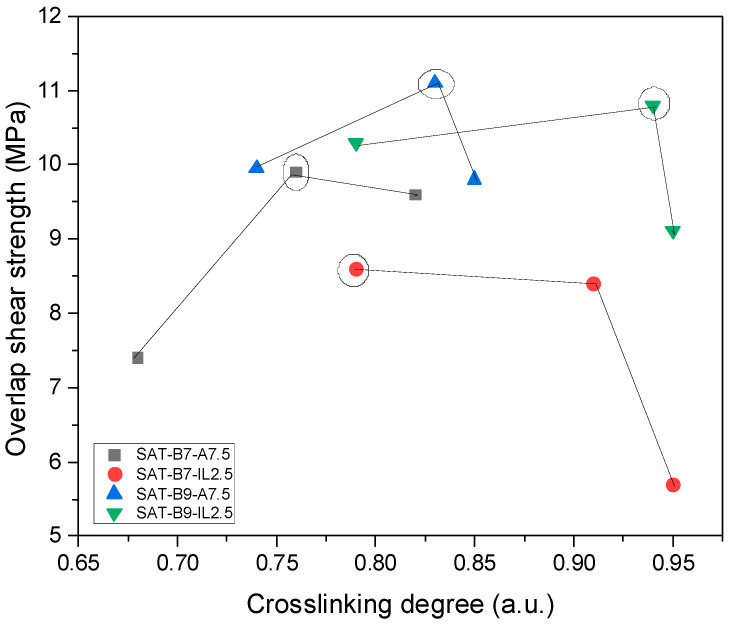
Dependence of the overlap shear strength of Al/SAT/Al joints on the cross-linking degree of SATs.

In general, α values are higher for thermally cured SATs with the ionic-liquid-type hardener (from 0.79 to 0.95 a.u.). However, α values for samples with the amine hardener are lower (from 0.69 to 0.82 a.u. for SAT-B7 and from 0.74 to 0.85 a.u. for SAT-B9). The lower value of the α parameter is probably caused by the denser polyacrylate network formed during the UV-cross-linking process in the presence of the amine-type additive. As we presented in [35], a too-high α value may deteriorate the shear strength of Al/SAT/Al overlap joints. Figure 12 shows the dependence of the overlap shear strength on the cross-linking degree value; the highest shear strength for each type of SAT is marked with a circle. 

It seems that the optimal cross-linking degree of SAT systems is 0.75–0.83 a.u. Additionally, DSC analyses revealed that adhesive films cured with the amine-type agent are characterized by two exothermic peaks (Figure 11). This applies to both thermally uncured SATs as well as thermally cured SATs under specific conditions. Interestingly, this effect was not observed in the case of EAC-B7-A and EAC-B9-A (Figure 2). Probably, this effect is related to the presence of poly(epoxyacrylate)-benzoxazine chains, created after the UV-cross-linking process (Figure 6 and Figure 7). Arguably, the position of the first peeks at a lower temperature (201 and 205 °C) corresponds to the exothermic polymerization peak of poly(epoxyacrylate)-benzoxazine chains. The second peak, with a higher temperature (ca. 309–322 °C), was assigned to the polymerization reaction of the remaining benzoxazine resin. Relatively higher τ values of SAT-B9 systems (especially SAT-B9-A7.5, i.e., 11.1 MPa) may be a result of the hydrogen interaction in the thermally cured SAT, between –OH of the phenolic structure of the polybenzoxazine and the oxygen of the carbonyl group (−C=O) of the UV-cross-linked EAC chain (Figure 8).

**Figure 13:**
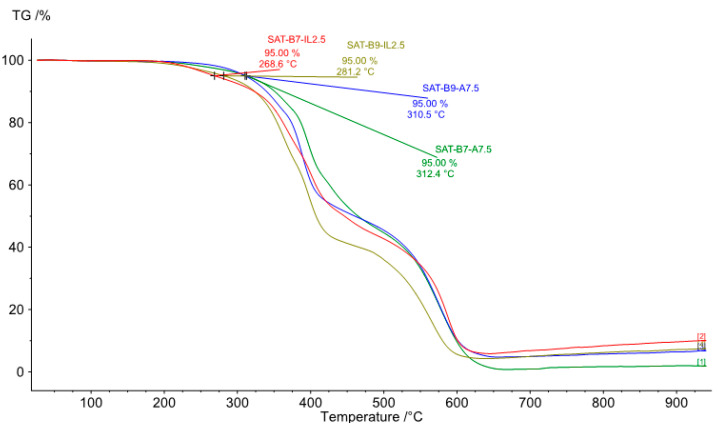
TGA curves under a N_2_ atmosphere of thermally cured SATs (195 °C for 60 min).

It has been proved, in numerous publications, that benzoxazine resins are characterized by high thermal stability. Figure 13 shows TGA thermograms of SATs thermally cured at 195 °C for 60 min. The type of hardener has a significant influence on the thermal stability of the cured SATs. It turns out that SATs with ionic liquids are characterized by a lower initial decomposition temperature T_d5_ (ca. 270 and 280 °C). However, samples with the amine hardener are characterized by a higher T_d5_ (310 °C for SAT-B9-A7.5 and 312 °C for SAT-B7-A-7.5). The study suggests that samples degrade in four stages: initial degradation up to ca. 310 °C, degradation between 300 and 400 °C, degradation between 400 and 600 °C, and the final degradation above 600 °C. Probably the first stage of degradation (300–400 °C) is related to the destruction of the polyacrylate structure and in the higher temperature range, to the degradation of the polybenzoxazine structure. The SAT-B9-IL2.5 system has the lowest thermal stability. As indicated, it is not homogeneous (Figure 3) and there is probably the formation of an insignificant common polyacrylate-benzoxazine network during the UV-cross-linking step. Thus, the low density of the polyacrylate network has a positive effect on the polymerization process of the BR. For this reason, the cured SAT-B9-IL2.5 has a high cross-linking degree (0.94 a.u).

This results in a relatively high content of a char yield at 800 °C. In contrast, the homogeneous SAT-B7-A.7.5 system is characterized by a higher thermal stability up to 500 °C. This is due to the generally higher cross-linking density of SAT-B7-A. As indicated earlier, this system forms a common polyacrylate-benzoxazine network after the UV-cross-linking step. For this reason, it has the lowest α value (0.76 a.u.) as polymerization of the benzoxazine is hindered in the presence of a dense polyacrylate-benzoxazine network and therefore the chart yield at 800 °C is the lowest. 

Finally, properties of the four selected SATs with benzoxazine resins were compared to SAT-0 (based on the EAC and the epoxy resin). The curing efficiency of thermosetting systems (Cure Index, CI) was also evaluated. The comparison is summarized in Table 6. 

Generally, UV-cross-linked SATs based on BRs are characterized by much higher adhesion to steel than SAT-0 based on the epoxy resin. The only exception is the SAT-B7-IL2.5 system (9.5 N/25 mm), for reasons we previously described. Higher adhesion for systems with BRs is due to the formation of hydrogen bonds between the –OH groups (from BRs) with the polar surface of the steel (to a greater extent than in the case of the epoxy resin). In turn, thermally cured SATs with BRs are characterized by higher thermal resistance (higher T_d50_ values) than the SAT-0 sample, as expected. However, the quality of thermal cross-linking (evaluated by the calculation of CI) for benzoxazine systems is generally good (G) for SAT-B7-A7.6, SAT-B7-IL2.5, and SAT-B9-A7.5 or excellent (E) for SAT-B9-IL2.5 in comparison to the epoxy resin system (SAT-0). This result corresponds to the overall higher shear strength values of adhesive joints with SAT-B7 and SAT-B9 than with SAT-0 (except for the SAT-B7-IL2.5 sample; 8.4 MPa); however, the influence of the LCA type should also be taken into account. The surprising result of the quality of cross-linking for SAT-B9-IL2.5 (E—excellent cure), although it does not correspond with mechanical properties after thermal curing (lower τ result; 10.8 MPa), confirms that the hardening process is different in this system, as previously mentioned (separate polyacrylate and polybenzoxazine networks). This can also be confirmed by the much lower thermal resistance of such system (T_d50_ only 407 °C).

## 4. Conclusions

In this paper, a new application of benzoxazine resins has been revealed, i.e., as a photoreactive and thermosetting component of adhesive films based on a photoreactive epoxyacrylate copolymer (self-adhesive structural tapes (SATs)). In addition, the possibility of using two types of polymerization initiators, both benzoxazine resins, for ring opening polymerization and cationic polymerization of an epoxyacrylate copolymer was investigated (i.e., amine type and ionic liquid type). At the beginning, the miscibility of systems and their pot-life were assessed. Only the adhesive composition based on the benzoxazine resin Araldite MT 35700 (B7) and the amine-type hardener (A233) was fully homogenous and characterized by the longest pot-life (30 days). The ionic-liquid-type hardener reduced the miscibility of epoxyacrylate copolymer-benzoxazine resin systems (EAC-BR) and additionally shortened their pot-life. The impact of BRs and the hardener type on the UV-cross-linking and the thermal curing process of SATs was analyzed. Generally, the UV-cross-linking process was more effective in a homogeneous system (with the B7 resin and the amine-type hardener), which was confirmed by the decrease in adhesion and an increase in cohesion values of SAT-B7-A with increase in the A233 content. It has been found that the amine hardener and the benzoxazine molecule may act similar to hydrogen donors for benzophenone moieties in the EAC chain, leading to the formation of a dense polyacrylate-benzoxazine network after the UV-cross-linking process. However, better adhesion to steel and tack was exhibited by SATs based on the B9 resin (even up to 47 N/25 mm), which results from low T_g_ values of such systems (below −20 °C). Additionally, aluminum joints with SATs containing 2.5, 5, or 7.5 wt. parts of the amine or ionic liquid initiator (after the thermal curing process at 180 °C) recorded higher shear strength for systems containing the B9 resin (8–10.3 MPa) than for those containing the B7 resin (5.2–9 MPa). The highest shear strength (11.1 MPa) was achieved for SAT-B9-A7.5 (at 195 °C for 60 min). Thermal analysis (DSC data) of thermally cured SATs revealed two exothermic processes in the case of SATs with the amine-type hardener. This confirms the partial inclusion of the benzoxazine resin in the cross-linked polyacrylate structure at the UV-cross-linking stage and the higher cross-linking density of such systems. The second peak of the exothermic reaction in DSC thermograms was attributed to the polymerization of the benzoxazine resin itself. Additional calculations based on DSC data allowed the evaluation of the efficiency of curing (Cure Index) in epoxyacrylate copolymer-benzoxazine resin systems compared to the system with the epoxy resin. These results correspond to the generally higher mechanical and thermal strength of benzoxazine systems (but only for homogeneous systems).

In summary, obtaining of UV-cross-linkable and thermally curable adhesive films with high adhesion and relatively high shear strength by using mixtures of the benzoxazine resin and the photoreactive epoxyacrylate copolymer and in the presence of amine hardener is possible. These types of adhesives could potentially be used in automotive and aviation industries to obtain mechanically and thermally durable metal adhesive joints.

## Figures and Tables

**Table 1:** Characteristic of tested benzoxazine resins [13].

BR Symbol	Trade Name	η at 120 °C (mPa·s)	T_g_ (°C)
B7	Araldite MT 35700	<1000	175
B9	Araldite MT 35910	2000–2500	165

**Table 2:** Dynamic viscosity, molecular weight, and glass transition temperature values of the EAC.

Sample	η (Pa·s)	Mn (g/mol)	Mw (g/mol)	T_g_ (°C)
EAC	12.7	135,500	436,800	−21

**Table 3:** Structural self-adhesive tape compositions containing different benzoxazine resins and latent curing agents.

SAT Acronym	EAC (wt. parts)	BR		LCA		MM	PI	AP
		Type	(wt. parts)	Type	(wt. parts)	(wt. parts)	(wt. part)	(wt. parts)
SAT-B7-A2.5	100	B7	50	A233	2.5	2	1	0.75
SAT-B7-A5					5			
SAT-B7-A7.5					7.5			
SAT-B7-IL2.5				IL	2.5			
SAT-B7-IL5					5			
SAT-B7-IL7.5					7.5			
SAT-B9-A2.5		B9	50	A233	2.5			
SAT-B9-A5					5			
SAT-B9-A7.5					7.5			
SAT-B9-IL2.5				IL	2.5			
SAT-B9-IL5					5			
SAT-B9-IL7.5					7.5			

MM—multifunctional monomer; PI—photoinitiator; AP—adhesion promoter.

**Table 4:** Thermal features of EAC, BR, and EAC-BR mixtures with LCA (2.5 wt. parts) and their pot-life.

Sample Symbol	Pot-Life (Days)	T_g_ (°C)	T_i_ (°C)	T_p_ (°C)	ΔH (J/g)
EAC	180	−31	-	-	-
B7	-	36	218	234	345
B9	-	8	209	224	391
EAC-B7-A	30	−12	140	214	176
EAC-B7-IL	29	−11	121	213	173
EAC-B9-A	24	−24	145	228	226
EAC-B9-IL	17	−28	117	203	199

**Table 5:** Thermal parameters of the thermally cured SATs and the cross-linking degree (DSC data).

SAT	Curing Conditions (°C/min)	T_g_ (°C)	T_i_ (°C)	T_p_ (°C)	ΔH (J/g)	α
SAT-B7-A7.5	before	−3	130	201/309	219	-
	180/60	-	195	224/317	67	0.69
	195/60	-	213	236/315	52	0.76
	195/90	-	229	245/317	39	0.82
SAT-B7-IL2.5	before	−12	124	209	211	-
	180/60	-	187	226	43	0.79
	195/60	-	219	249	18	0.91
	195/90	-	221	249	10	0.95
SAT-B9-A7.5	before	−21	138	205/322	231	-
	180/60	-	194	229/322	60	0.74
	195/60	-	213	237/322	39	0.83
	195/90	-	228	242/321	35	0.85
SAT-B9-IL2.5	before	−25	117	203	185	-
	180/60	-	192	238	38	0.79
	195/60	-	215	245	10	0.94
	195/90	-	220	249	9	0.95

**Table 6:** Comparison of adhesive, mechanical, and thermal properties, as well as CI values, of selected thermosetting systems based on the EAC.

Thermoset System	Adhesion to Steel ^(2)^ (N/cm^2^)	τ ^(3)^ (MPa)	α (a.u.)	T_d50_ (°C)	ΔH	ΔT	CI
SAT-0 ^(1)^	11.7	9.3	0.95	404	-	-	-
SAT-B7-A7.5	21.0	9.9	0.76	465	1.06	1.70	1.75 (G)
SAT-B7-IL2.5	20.3	8.4	0.91	446	1.03	1.37	1.41 (G)
SAT-B9-A7.5	35.0	11.1	0.83	460	1.14	1.71	1.95 (G)
SAT-B9-IL2.5	9.5	10.8	0.94	407	0.9	1.37	1.23 (E)

^(1)^ data from [35]; ^(2)^ adhesion to steel of UV-cross-linked SATs (before thermal curing); ^(3)^ overlap shear strength of Al/SAT/Al joints thermally cured for 60 min at 160 °C (SAT-0) or 195 °C (SAT-B7 and SAT-B9); G—good cure; E—excellent cure.

## Data Availability

Not applicable.
